# Audiological and electrophysiological alterations in HIV-infected individuals subjected or not to antiretroviral therapy^☆^

**DOI:** 10.1016/j.bjorl.2017.07.003

**Published:** 2017-08-02

**Authors:** Carla Gentile Matas, Alessandra Giannella Samelli, Fernanda Cristina Leite Magliaro, Aluisio Segurado

**Affiliations:** a Universidade de São Paulo (USP), Faculdade de Medicina, Departamento de Fisioterapia, Fonoaudiologia e Terapia Ocupacional, São Paulo, SP, Brazil; b Universidade de São Paulo (USP), Faculdade de Medicina, Departamento de Moléstias Infecciosas e Parasitárias, São Paulo, SP, Brazil

**Keywords:** Acquired immunodeficiency syndrome, Adults, Auditory evoked potentials, Hearing loss, HIV, Síndrome da imunodeficiência adquirida, Adultos, Potenciais auditivos evocados, Perda auditiva, HIV

## Abstract

**Introduction:**

The Human Immunodeficiency Virus (HIV) and infections related to it can affect multiple sites in the hearing system. The use of High Activity Anti-Retroviral Therapy (HAART) can cause side effects such as ototoxicity. Thus, no consistent patterns of hearing impairment in adults with Human Immunodeficiency Virus / Acquired Immune Deficiency Syndrome have been established, and the problems that affect the hearing system of this population warrant further research.

**Objectives:**

This study aimed to compare the audiological and electrophysiological data of Human Immunodeficiency Virus-positive patients with and without Acquired Immune Deficiency Syndrome, who were receiving High Activity Anti-Retroviral Therapy, to healthy individuals.

**Methods:**

It was a cross-sectional study conducted with 71 subjects (30–48 years old), divided into groups: Research Group I: 16 Human Immunodeficiency Virus-positive individuals without Acquired Immunodeficiency Syndrome (not receiving antiretroviral treatment); Research Group II: 25 Human Immunodeficiency Virus-positive individuals with Acquired Immunodeficiency Syndrome (receiving antiretroviral treatment); Control Group: 30 healthy subjects. All individuals were tested by pure-tone air conduction thresholds at 0.25–8 kHz, extended high frequencies at 9–20 kHz, electrophysiological tests (Auditory Brainstem Response, Middle Latency Responses, Cognitive Potential).

**Results:**

Research Group I and Research Group II had higher hearing thresholds in both conventional and high frequency audiometry when compared to the control group, prolonged latency of waves I, III, V and interpeak I–V in Auditory Brainstem Response and prolonged latency of P300 Cognitive Potential. Regarding Middle Latency Responses, there was a decrease in the amplitude of the Pa wave of Research Group II compared to the Research Group I.

**Conclusions:**

Both groups with Human Immunodeficiency Virus had higher hearing thresholds when compared to healthy individuals (group exposed to antiretroviral treatment showed the worst hearing threshold) and seemed to have lower neuroelectric transmission speed along the auditory pathway in the brainstem, subcortical and cortical regions.

## Resumo

**Introdução:**

O HIV e as infecções relacionadas a ele podem afetar vários locais do sistema auditivo. O uso de terapia antirretroviral altamente ativa pode causar efeitos colaterais, como ototoxicidade. Assim, não foram estabelecidos padrões consistentes de deficiência auditiva em adultos com HIV/Aids e os problemas que afetam o sistema auditivo dessa população justificam pesquisas futuras.

**Objetivos:**

Este estudo teve como objetivo comparar os dados audiológicos e eletrofisiológicos de pacientes HIV positivos com e sem Aids que recebiam terapia antirretroviral altamente ativa com os de indivíduos saudáveis.

**Método:**

Estudo transversal com 71 indivíduos (30-48 anos), dividido em grupos: Grupo de Pesquisa I: 16 indivíduos HIV-positivos sem Aids (não recebendo tratamento antirretroviral); Grupo de Pesquisa II: 25 indivíduos HIV-positivos com Aids (recebiam tratamento antirretroviral); Grupo Controle: 30 indivíduos saudáveis. Todos os indivíduos foram testados para limiares de condução aérea de tons puros a 0,25-8 kHz, altas frequências de 9-20 kHz, testes eletrofisiológicos (potencial evocado auditivo de tronco encefálico, potencial evocado auditivo de média latência, potencial cognitivo).

**Resultados:**

Os grupos de pesquisa I e II apresentaram limiares auditivos mais elevados em audiometria convencional e nas frequências altas quando comparados com o grupo controle, latência prolongada das ondas I, III, V e interpico I-V em resposta auditiva de tronco encefálico e latência prolongada de P300. Em relação às respostas de latência média, houve uma diminuição na amplitude da onda Pa do Grupo de pesquisa II em comparação com o grupo de pesquisa I.

**Conclusões:**

Ambos os grupos com HIV apresentaram limiares auditivos mais elevados quando comparados aos indivíduos saudáveis (o grupo exposto ao tratamento antirretroviral apresentou o pior limiar auditivo) e parecem ter menor velocidade de transmissão neuroelétrica ao longo da via auditiva nas regiões do tronco encefálico, subcortical e cortical.

## Introduction

The human immunodeficiency virus (HIV) causes Acquired Immune Deficiency Syndrome (AIDS), the devastating pandemic that continues to affect millions of people worldwide.^1^, ^2^

HIV infection and AIDS are distinct nosological entities. Many HIV-infected individuals present a normal number of immune cells, remaining asymptomatic for long periods of time, and could not be categorized as presenting the clinical definition of AIDS. To be clinically defined as AIDS, seropositive individuals older than 13 years of age, should present a CD4+ T lymphocyte count below 350 cells per mm^3^ (Ministério da Saúde, 1999)^3^ or develop at least one clinical condition that is consistent with AIDS.^4^

Since the advent of new antiretroviral drugs, there was a consistent shift in the treatment of HIV infection, providing to infected individuals a delay in the disease development and improving their clinical condition, although doubts regarding the toxic action of antiretroviral drugs on both peripheral and central auditory systems.

Until the 1990s, the most common treatment performed in antiretroviral therapy was the monotherapy, that is, the use of only one drug. Due to the evolution of the treatment, the combined therapy (better known as Highly Active Antiretroviral Therapy – HAART)^5^, ^6^ was established, and showed better prognosis for patients due to the inhibition of HIV replication, thus, it increased survival indexes and, consequently, provided mortality reduction, and is currently used in the treatment of individuals with AIDS.^7^

AIDS and the therapies developed to combat it have many side effects. Specifically, auditory and vestibular disorders afflict 5–34% of adults with HIV/AIDS.^8^ Hearing loss affects approximately 20–50% of patients with HIV/AIDS, and 75% of adults with AIDS have some kind of hearing disorder.^9^

The HIV and infections related to it can affect multiple sites in the hearing system causing observable abnormalities in patients including altered tympanograms, as well as threshold audiograms and auditory brainstem responses. Sensorineural hearing loss associated with HIV/AIDS may result from Central Nervous System (CNS) neoplasms, from ototoxic drug administration,^6^, ^9^, ^10^, ^11^, ^12^, ^13^, ^14^ from the effects of HIV on the CNS or on the peripheral auditory nerve, or from opportunistic infections.^15^ Individuals with HIV/AIDS also often have external otitis and otitis media.^16^

Despite these factors, no consistent patterns of hearing impairment in adults with HIV/AIDS have been established.^17^ Thus, the problems that affect the hearing system of this population warrant further research. The purpose of the present study was to compare the audiological and electrophysiological data of HIV-positive patients with and without AIDS, who were receiving HAART, to healthy individuals.

## Methods

### Sample characteristics

This cross-sectional, observational and descriptive study was conducted at the Laboratory for Hearing Research in Auditory Evoked Potentials of the Speech and Hearing Sciences Department of the Faculdade de Medicina da Universidade de São Paulo (FMUSP) between 2012 January and 2014 December. Research methods were approved by the Ethics Committee for Analysis of Research Projects (CAPPesq) of the Clinical Board of Hospital das Clínicas and FMUSP under protocol number 1026/04. All participants signed an informed consent form. This study had been carried out in accordance with Declaration of Helsinki.

The sample consisted of 71 individuals between the ages of 30 and 48, divided into three groups. The Research Group I (RGI) included 16 HIV-positive patients without AIDS, whose status were confirmed by serology and who had never received any antiretroviral treatment. The Research Group II (RGII) included 25 HIV-positive patients with AIDS, whose status were confirmed by serology. All patients in RGII were receiving HAART (combination therapy), consisting of at least three of the following drugs: lamivudine, zidovudine, efavirenz, didanosine, nevirapine, lopinavir-r, tenofovir, stavudine, indinavir, abacavir, amprenavir, ritonavir, and atazanavir.

Finally, the Control Group (CG) consisted of 30 healthy subjects with reported and confirmed HIV-negative status, no history of psychiatric and neurological disease, no hearing, language, or auditory processing complaints.

Individuals in the RGI and RGII were referred to the study by the House of AIDS – Zerbini Foundation (Casa da AIDS – Fundação Zerbini, São Paulo, Brazil) and the City Health Services Specialized in Sexually Transmitted Diseases (STD/AIDS) of the City of São Paulo Health Department.

Exclusion criteria for all three groups were: pure tone audiometry from moderately severe to profound hearing loss, pregnancy, presence of opportunistic infections in activity, history of otologic surgery or history of non-HIV-related disease, presence of any cognitive impairment that could affect hearing tests results (these data were obtained in the medical records).

### Procedures

Data regarding HIV infection, exposure category, as well as history of use of antiretroviral and other drugs with ototoxic potential were obtained from the medical records of patients in RGI and RGII. Interviews were conducted to assess the presence of risk indicators for hearing loss. Visual inspection of the external ear canal (Heine otoscopy) was performed to identify any possible obstructions by cerumen or foreign bodies that could interfere with the hearing tests.

Screening immittance measurements (tympanometry, acoustic reflex) were carried out with AT 235 (Interacoustic) to verify the middle ear conditions. Pure-tone air conduction thresholds at all frequencies from 0.25 to 8 kHz and in the extended high frequencies at 9, 10, 12.5, 14, 16, 18 and 20 kHz were carried out with a GSI 61 Clinical Audiometer (Grason-Stadler, Inc., Madison, WI) using standard audiometric techniques in a sound-attenuated testing room.

A classification to determine the grade of hearing loss and to classify the type of hearing loss was used: conductive, sensorineural, mixed or isolated hearing loss in high frequencies.^18^, ^19^

The electrophysiological tests (Auditory Brainstem Response – ABR; Middle Latency Responses – MLR and Cognitive Potential – P300) were carried out in an electric- and sound-attenuated testing room. Electrophysiological evaluation was made using a two-channel electroneuromyograph (Express Traveler Portable System; Biological Systems Corp., Mundelein, IL, USA). Standard Bio-logic TDH-39 phones were used to deliver the sound stimuli for the electrophysiological tests. Electrodes were placed on the forehead (Fpz), left and right mastoids (M1 and M2), and left and right temporal-parietal junctions (C3 and C4) according to the standard International Electrode System (IES). Impedance values were maintained below 5 kΩ.

To ABR testing, a rate of 19 clicks per second with 0.1 μs duration was used with a filter slope of 12 dB/octave, with the high filter setup at 100 Hz and the low filter at 1500 Hz and 2000 sweeps. The stimulus was 80 dBnHL. ABR measurements were duplicated to ensure fidelity. The absolute latencies of waves I, III and V, and interpeaks I–III, III–V, I–V were analyzed.

The MLR was obtained with a monaural click presented at 70 dBnHL at a rate of 9.9 clicks per second, with a 10–300 Hz band-pass filter, for a total of 1000 stimuli. Na and Pa wave latencies and Na–Pa amplitudes were obtained contra-laterally (C3/A2, C4/A1) and ipsi-laterally (C3/A1, C4/A2).

Individuals were asked to remain with their eyes closed during the recording of Cognitive Potentials – P300, to control the eye-movement artifacts. The oddball paradigm was used in P300 recordings. This paradigm was based on distinguishing between a target stimulus repeated randomly (20% of the time) and the non-target stimulus with frequent repetition (80% of the time). Subjects were asked to count the target stimuli whenever they discriminated them. Monaural auditory stimulus was presented. Frequencies were 1000 Hz for the frequent stimulus (non-target) and 1500 Hz for the rare (target) stimulus. The stimulus was set at 75 dBnHL. A rate of 1.1 tone-burst per second was used with the low filter setup at 30 Hz and the high filter at 1 Hz and 300 sweeps. For the P300, non-target stimuli were subtracted from target stimuli and the latency was measured at the highest positive point (amplitude) from 250 to 650 ms.

Component analysis of the electrophysiological tests was performed by the lead researcher and by a second experienced researcher in the field of electrophysiology. The electrophysiological recordings were evaluated blindly; the researchers did not know about the association participant/group.

Results were sent to the care provider institutions of the individuals in RGI and RGII. In case of abnormal results, patients were referred for ENT evaluation and instructed to return for reassessment after three months.

Statistical analyses were conducted with ANOVA (one factor), Tukey test and Fisher's exact test. We also calculated odds ratio and descriptive measures. Initially, the left and right ears of each group were compared for each test. As no differences were found, ears were grouped and then compared. A *p*-value of 0.05 was considered significant, and was designated with an asterisk (*).

## Results

Table 1 shows the distribution of gender and age in CG, RGI and RGII; hearing complaints, CD4+ T lymphocyte counts and time of HIV infection in RGI and RGII groups. There were no statistically significant differences between groups regarding gender (*p* = 0.218), age (*p* = 0.119), hearing complaints (*p* = 0.064), CD4+ T lymphocyte counts (*p* = 0.193) and time of HIV infection (*p* = 0.168).

**Table 1:** Descriptive analysis to gender, age, presence of hearing complaints, CD4+ T lymphocyte counts and time of HIV infection of CG, RGI and RGII.

	CG	RGI	RGII
Female:male	14:16	4:14	9:18
Age^a^	36.6 ± 6.1	39.4 ± 8.1	40.3 ± 6.7
Hearing complaints (%)	–	61	89
CD4+ T lymphocyte counts^b^	–	585.3 ± 242.0	477.0 ± 273.3
Time of HIV infection^c^		86.5 ± 57.7	111.6 ± 57.5

a In years (mean ± standard deviation).

b Cells per mm^3^.

c In months.

**Figure 1:**
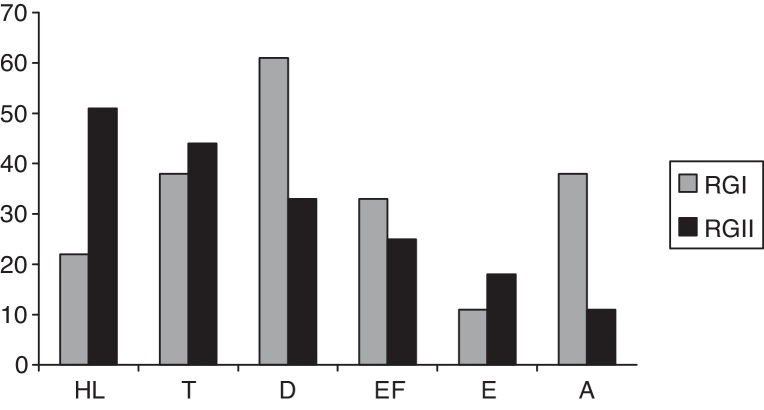
Hearing complaints (in %) in HIV-positive individuals of RGI and RGII (HL, hearing loss; T, tinnitus; D, dizziness; EF, ear fullness; E, earache; A, asymptomatic).

Fig. 1 shows the hearing complaints in HIV-positive individuals of RGI and RGII. The most frequent complaint was dizziness, followed by tinnitus in RGI. In RGII, the most frequent complaint was hearing loss, followed by tinnitus. No significant differences were observed among RGI and RGII groups (*p* = 0.064); 61% of individuals in the RGI group and 89% of the RGII had at least one complaint of hearing.

Table 2 shows the percentage of hearing loss type found in each group. Sensorineural represented the most common type of hearing loss in the three groups. The RGII had a higher percentage of hearing loss (44%), followed by the RGI with 25%. When RGI and RGII were compared to the CG regarding the risk of hearing loss, RGII had a risk rate and odds ratio significantly greater than RGI (Table 3).

**Table 2:** Absolute and relative hearing loss frequency in RGI, RGII and CG.

Type of hearing loss	RGI (*n* = 16)			RGII (*n* = 25)			CG (*n* = 30)		
	A	B	C	A	B	C	A	B	C
*n* (%)	1 (6.25%)	3 (18.75%)	0 (0%)	4 (16%)	5 (20%)	2 (8%)	0 (0%)	2 (6.7%)	0 (0%)

A, conductive hearing loss; B, sensorineural hearing loss or isolated hearing loss in high frequencies; C, mixed loss.

**Table 3:** Comparison of the risks of hearing loss among RGI, RGII and CG.

	RGI vs. CG	RGII vs. CG
Rate (CG rate = 0.066)	0.25	0.44
Risk ratio (CI)	3.75 (0.76–18.30)	6.6 (1.61–27.03)
Odds ratio (CI)	4.66 (0.75–29)	11 (2.13–56.56)
*p*-value (Fisher's exact test)	0.198	0.002

The average hearing thresholds in conventional and high frequency audiometry were lower for CG than observed in the other two groups (Table 4). The differences in hearing thresholds between CG and RGII were statistically significant for all evaluated frequencies. Comparison between CG and RGI showed differences in threshold that were significant and starting at a frequency of 6 kHz. In the comparison between RGI and RGII, significant differences were not found only for the frequencies of 8 and 9 kHz.

**Table 4:** Mean hearing thresholds by frequency in RGI, RGII and CG.

		Mean (dBHL)	SD	*p*-value	Tukey test
250 Hz	RGI	10.15	11.39	<0.0001^a^	M1 vs. M2, *p* < 0.01^a^
	RGII	25.3	23.69		M1 vs. M3, non-significant
	CG	5.5	4.08		M2 vs. M3, *p* < 0.01^a^
500 Hz	RGI	8.28	9.88	<0.0001^a^	M1 vs. M2, *p* < 0.01^a^
	RGII	22.9	23.34		M1 vs. M3, non-significant
	CG	5.16	3.9		M2 vs. M3, *p* < 0.01^a^
1000 Hz	RGI	7.34	9.24	<0.0001^a^	M1 vs. M2, *p* < 0.01^a^
	RGII	21	23.66		M1 vs. M3, non-significant
	CG	5.16	4.69		M2 vs. M3, *p* < 0.01^a^
2000 Hz	RGI	6.09	11.41	<0.0001^a^	M1 vs. M2, *p* < 0.01^a^
	RGII	20.8	22.64		M1 vs. M3, non-significant
	CG	4	5.02		M2 vs. M3, *p* < 0.01^a^
3000 Hz	RGI	9.21	9.25	<0.0001^a^	M1 vs. M2, *p* < 0.01^a^
	RGII	22.6	21.97		M1 vs. M3, non-significant
	CG	3.83	4.25		M2 vs. M3, *p* < 0.01^a^
4000 Hz	RGI	12.65	11.49	<0.0001^a^	M1 vs. M2, *p* < 0.01^a^
	RGII	25.5	24.27		M1 vs. M3, non-significant
	CG	4.91	5.48		M2 vs. M3, *p* < 0.01^a^
6000 Hz	RGI	17.34	12.88	<0.0001^a^	M1 vs. M2, *p* < 0.01^a^
	RGII	29.1	28.61		M1 vs. M3, *p* < 0.05^a^
	CG	6.25	6.55		M2 vs. M3, *p* < 0.01^a^
8000 Hz	RGI	19.06	21.19	<0.0001^a^	M1 vs. M2, non-significant
	RGII	27.5	29.19		M1 vs. M3, *p* < 0.01^a^
	CG	5.25	5.85		M2 vs. M3, *p* < 0.01^a^
9000 Hz	RGI	23	21.87	<0.0001^a^	M1 vs. M2, non-significant
	RGII	32.73	30.24		M1 vs. M3, *p* < 0.01^a^
	CG	9.25	7.52		M2 vs. M3, *p* < 0.01^a^
10,000 Hz	RGI	20.83	21.77	<0.0001^a^	M1 vs. M2, *p* < 0.05^a^
	RGII	32.73	29.45		M1 vs. M3, *p* < 0.05^a^
	CG	7.83	8.09		M2 vs. M3, *p* < 0.01^a^
11,200 Hz	RGI	24	23.28	<0.0001^a^	M1 vs. M2, *p* < 0.01^a^
	RGII	39.04	28.90		M1 vs. M3, *p* < 0.01^a^
	CG	8.66	9.56		M2 vs. M3, *p* < 0.01^a^
12,500 Hz	RGI	30	27.32	<0.0001^a^	M1 vs. M2, *p* < 0.05^a^
	RGII	43.69	30.50		M1 vs. M3, *p* < 0.01^a^
	CG	11.25	12.13		M2 vs. M3, *p* < 0.01^a^
14,000 Hz	RGI	34.33	26.61	<0.0001^a^	M1 vs. M2, *p* < 0.01^a^
	RGII	53.69	24.42		M1 vs. M3, *p* < 0.01^a^
	CG	12.5	14.51		M2 vs. M3, *p* < 0.01^a^
16,000 Hz	RGI	34	23.64	<0.0001^a^	M1 vs. M2, *p* < 0.01^a^
	RGII	50.59	14.53		M1 vs. M3, *p* < 0.01^a^
	CG	12.91	14.70		M2 vs. M3, *p* < 0.01^a^
18,000 Hz	RGI	20.16	13.16	<0.0001^a^	M1 vs. M2, *p* < 0.01^a^
	RGII	32.38	8.05		M1 vs. M3, *p* < 0.01^a^
	CG	10.16	9.78		M2 vs. M3, *p* < 0.01^a^
20,000 Hz	RGI	7.85	7.98	<0.0001^a^	M1 vs. M2, *p* < 0.01^a^
	RGII	13.92	7.03		M1 vs. M3, *p* < 0.01^a^
	CG	2.41	4.26		M2 vs. M3, *p* < 0.01^a^

SD, standard deviation; *p*-value and Tukey test compared hearing thresholds of RGI, RGII and CG.

a Indicates statistically significant *p*-value.

Table 5 compares ABR components in the three groups. The CG displayed the shorter latencies, and significant differences among groups were detected for waves I, III and V and for interpeak interval I–V. Regarding medium-latency components, significant differences in latency and amplitude were found for Na and Pa waves only for the electrode positioned at C3 (Table 6). Finally, Table 6 indicates also that, regarding the P3 wave, CG had shorter latency then RGI and RGII, which were similar to each other.

**Table 5:** Mean latency of waves I, III and V, as well as interpeaks I–III, III–V and I–V obtained with ABR from individuals in RGI, RGII and CG.

Latencies	Wave I			Wave III			Wave V		
	RGI	RGII	CG	RGI	RGII	CG	RGI	RGII	CG
Mean (ms)	1.82	1.73	1.55	3.87	3.93	3.74	5.89	5.94	5.67
SD	0.52	0.39	0.10	0.30	0.51	0.15	0.33	0.58	0.16
*p*-value	0.001^a^			0.018^a^			0.001^a^		
Tukey test	M1 vs. M2, non-significant			M1 vs. M2, non-significant			M1 vs. M2, non-significant		
	M1 vs. M3 *p* < 0.01			M1 vs. M3, non-significant			M1 vs. M3, *p* < 0.05		
	M2 vs. M3 *p* < 0.05			M2 vs. M3, *p* < 0.05			M2 vs. M3, *p* < 0.01		

Interpeaks	I–III			I–V			III–V		
	RGI	RGII	CG	RGI	RGII	CG	RGI	RGII	CG
Mean (ms)	2.29	2.23	2.19	4.39	4.33	4.05	2.01	2.09	2
SD	0.38	0.31	0.12	0.62	0.62	0.41	0.14	0.38	0.41
*p*-value	0.265			0.005^a^			0.400		
Tukey test	–			M1 vs. M2, non-significant			–		
				M1 vs. M3, *p* < 0.01					
				M2 vs. M3, non-significant					

SD, standard deviation; *p*-value and Tukey test compared latencies of RGI, RGII and CG.

a Indicates statistically significant *p*-value.

**Table 6:** Mean latencies and amplitudes of Na and Pa waves for MLR and mean latencies for P300 waves in RGI, RGII and CG.

Latencies	Na (C3)			Pa (C3)			Na (C4)			Pa (C4)		
	RGI	RGII	CG	RGI	RGII	CG	RGI	RGII	CG	RGI	RGII	CG
Mean (ms)	21.37	21.50	19.26	35.03	32.35	34.12	19.73	20.96	19.58	32.37	32.13	34.33
SD	4.04	6.21	3.06	4.81	6.45	3.15	1.95	6.21	3.91	4.39	6.97	3.62
*p*-value	0.022^a^			0.039^a^			0.255			0.059^b^		
Tukey test	M1 vs. M2, non-significant			M1 vs. M2, *p* < 0.05			–			–		
	M1 vs. M3, non-significant			M1 vs. M3, non-significant								
	M2 vs. M3, non-significant			M2 vs. M3, non-significant								

Amplitudes	C3			C4		
	RGI	RGII	CG	RGI	RGII	CG
Mean (μv)	3.50	1.59	2.31	1.78	1.66	2.04
SD	3.01	1.38	2.70	1.28	1.14	1.92
*p*-value	0.002^a^			0.421		
Tukey test	M1 vs. M2, *p* < 0.01			–		
	M1 vs. M3, non-significant					
	M2 vs. M3, non-significant					

Latencies	P300		
	RGI	RGII	CG
Mean (ms)	337.5	331.6	313.23
SD	28.12	42.98	30.06
*p*-value	0.002^a^		
Tukey test	M1 vs. M2, non-significant		
	M1 vs. M3, *p* < 0.01		
	M2 vs. M3, *p* < 0.05		

SD, standard deviation; *p*-value and Tukey test compared latencies of RGI, RGII and CG.

a Indicates statistically significant *p*-value.

b Indicates marginal statistical significance. Na waves obtained with C3 and C4 electrode positions and Pa with C3 and C4 electrode positions. Na-Pa amplitude obtained with C3 and C4 electrode positions.

## Discussion

The frequent occurrence of hearing abnormalities in individuals with HIV/AIDS has been long known,^20^, ^21^ and many potential targets have been reported, from the middle ear to the central nervous auditory system. However, a discernible pattern has not been found, nor has a primary pathology that may result in hearing impairment.^17^ Thus, the present study focused on these issues by simultaneously assessing the peripheral and central auditory pathway in individuals with HIV/AIDS, subjected or not to HAART, and in healthy individuals.

Regarding hearing complaints, no significant differences were observed among RGI and RGII groups; 61% of individuals in the RGI group and 89% of the RGII had at least one complaint of hearing. Several studies reported an association between HIV infection and signs/symptoms neurotological. Individuals with AIDS often have hearing complaints, because the ENT manifestations are common at any stage of the disease, leading to specific symptoms such as hearing loss, tinnitus, dizziness and ear fullness,^13^, ^14^, ^20^, ^21^, ^22^ which also were observed in the present study.

Sensorineural hearing loss represented the most common type in the three groups, which is in agreement with previous studies.^9^ The RGII displayed the highest percentage loss (44%), followed by RGI (25%). These findings agree with previous work showing losses between 21% and 49%.^23^ It is worth mentioning that these losses may vary depending on the criteria adopted for hearing loss and the age range included in each study.^9^, ^15^, ^17^

We also observed an increased risk for hearing loss in RGII. We found a risk ratio of 6.6 for RGII, almost double the one found for RGI (3.75). The odds ratio for the presence of hearing loss was 11 for RGII and 4.66 for RGI in comparison with the CG. These finding agree with a previous work showing that HIV-positive individuals subjected to HAART had higher odds ratio for hearing loss than healthy individuals. However, these authors did not make comparisons with HIV-positive individuals who did not receive HAART. Our results contrast with those reported previously^16^ that found a weak positive association between HIV status and poor cochlear function, with an odds ratio slightly higher than one. However, this previous work relied on a different technique (otoacoustic emissions) and did not separate individuals regarding their HAART status.

The CG had lower hearing thresholds than the other two groups, and RGI had lower thresholds than RGII with significant differences in a majority of the frequencies tested. These findings suggest the existence of mechanisms that underlie the worsening of hearing thresholds in the two groups of patients with HIV/AIDS: viral presence in the cochlea previously^24^; a combination of HIV with opportunistic infections; and/or the effects ototoxic therapeutic agents.^2^, ^9^, ^15^, ^17^ Specifically in RGII (individuals with AIDS), these mechanisms may act in synergy, whereas the non-drug administration of HAART in the RGI suggests the absence of the effects of ototoxic drugs specific to AIDS. This hypothesis is supported by work using HEI-OC1 cells that evaluated the ototoxic potential of 14 anti-HIV agents, a majority of which was used by RGII patients.^6^ In this previous study, the authors suggest that many of these drugs, used as antiretroviral agents, may have deleterious effects for the auditory system of patients and further research is under way to validate this idea.

It is worth noting that sensorineural hearing loss more often affects patients with severe HIV infection.^22^ This fact may also explain why RGI individuals, who were not on HAART and likely had less severe infections than RGII, had less intense hearing problems.

Regarding ABR, the CG had shorter latencies than RGI and RGII, which were similar between them. Other previous studies have compared the ABR of HIV-positive patients and controls.^25^, ^26^, ^27^, ^28^, ^29^, ^30^, ^31^ Results from these reports included latency delays of one or more waves, and increased latencies of one or more interval interpeaks, all of which agree with the present study.

HIV can affect the subcortical and cortical areas of the CNS, and the ABR-generating system depends on the temporal synchronization of neuronal activity. Thus, the central conduction of auditory information in patients with various degrees of HIV infection may be affected, which can generate changes to ABR waves, often causing increased latencies of waves III and V, whereas waves I and II maintain normal absolute latencies.^32^

Regarding ABR in individuals with HIV/AIDS, these results indicate the usefulness of this procedure for the detection of early signs of neurodegeneration in this population, and in monitoring how fast lesions evolve.^25^, ^28^ These findings also emphasize the role of immunosuppression in the development of neural abnormalities involving the brainstem auditory pathway during the course of disease.^26^

Regarding MLR, we observed significant differences in latency and amplitude of Na and Pa waves with the electrode positioned at C3. In two other studies using MLR in HIV/AIDS patients, one found a tendency toward the increase in latency and reduction in amplitude of the PA wave,^33^ whereas the other found ear and electrode effects happening concomitantly.^31^ The two studies suggested that auditory information was impaired in cortical and subcortical regions, reinforcing the need for the detailed investigation of auditory function in individuals with HIV/AIDS.

This result may be explained by the fact that the first CNS alteration in this population includes subcortical demyelination, even before clinical neurological manifestations are present.^34^ Along the same line, other work suggested that HIV affects subcortical and cortical CNS areas, which are crucial for the integrity of MLR wave generation, thus explaining abnormalities.^32^ Nevertheless, studies that assess the MLR of HIV-positive individuals remain scant, as most research utilizes ABR and P300.

Concerning P300, we found significant differences among the three groups for the P3 wave, with shorter latencies the CG and no differences between RGI and RGII. Previous studies have used long latency auditory evoked potentials to assess the central auditory pathway of individuals with HIV/AIDS.^31^, ^33^, ^35^, ^36^, ^37^, ^38^, ^39^ All of these studies reported alterations, mainly an increase in latency or reduction of amplitude of the waves N1, P2 and/or P3, in the comparison with healthy subjects. These findings may be associated with impaired cognition, a problem that may be present in AIDS patients.^35^, ^37^ Other authors added that P300 provides an early indicator of cognitive deficits in seropositive patients, for whom reduced amplitude suggests lower attention, and longer latency a slower processing of information.^38^, ^39^

The novel approach of the present study was to simultaneously evaluate the peripheral and central auditory pathways of HIV-positive individuals subjected or not to HAART, and to compare them to healthy individuals. Our findings suggest that HIV-positive individuals may develop alterations of the peripheral and central auditory systems that may result from direct viral action, from the presence of opportunistic infections, and/or from the use of ototoxic drugs.

## Limitations

Regarding the number of individuals, it is worth mentioning that some challenges were found to enlarge the sample, especially in relation to the referral of the RGI individuals, once the HIV positive population who do not underwent antiretroviral treatment is scarce. For this reason, it was not possible to achieve the same number of individuals in the RGII.

Specifically concerning RGII, since each patient received at least three medications among those available for HAART, it was not possible to obtain a homogeneous sample as to the type of medication used.

## Conclusions

HIV-positive patients with and without AIDS, who were receiving HAART, when compared to healthy individuals, presented:

- Elevated hearing thresholds in behavioral hearing evaluations (Conventional Tonal Audiometry and High Frequency Audiometry), where the group subjected to HAART had the highest thresholds.
- Longer latencies regarding waves I, III and V as well as interpeak I–V for ABR, suggesting lower speeds of neuroelectric impulse transmission throughout the brainstem auditory pathway.
- Longer P300 latency, suggesting reduced processing speed of auditory information in cortical regions.
- A reduction in the amplitude of the Pa wave in MLR in the comparison of RGII with RGI, suggesting alterations to the auditory pathway in cortical and subcortical regions.

## Conflicts of interest

The authors declare no conflicts of interest.
